# Experimental study on the effect of CO_2_ injection on the permeability characteristics of reservoir sandstone

**DOI:** 10.1371/journal.pone.0317134

**Published:** 2025-01-22

**Authors:** Bing Liang, Yangqi Ma, Weiji Sun, Xu Qin, Guotao Shi, Bo Liang

**Affiliations:** School of Mechanics and Engineering, Liaoning Technical University, Fuxin, Liaoning, China; Shenyang Jianzhu University, CHINA

## Abstract

Using the Ordos Basin dry sandstone and sandstone saturated with different saline concentrations as research subjects, a self-developed constant temperature and pressure CO_2_ injection simulation device was employed to conduct permeability tests on sandstone under varying effective stresses and CO_2_ injection pressures. The test results indicated that during the CO_2_ injection process, the permeability of dry sandstone was two orders of magnitude higher than that of sandstones saturated with different saline concentrations. When the effective stress increases from 10 MPa to 28 MPa, the fissure compressibility of reservoir sandstone is influenced by the saturation of different saline concentrations, with the compressibility coefficients for 0%, 15%, and 30% saline-saturated sandstone being 0.00495 MPa⁻^1^, 0.00614 MPa⁻^1^, and 0.01879 MPa⁻^1^, respectively. The primary reasons for the reduced permeability of sandstone are as follows: supercritical CO_2_ lowers the mechanical properties of sandstone; high-concentration saline induces crystallization within the sandstone, resulting in a blockage effect. High-concentration saline increases the fissure compressibility of sandstone, decreases the permeability of the sandstone reservoir, and ultimately affects the injectability of the CO_2_ in the reservoir.

## 1. Introduction

The international community has proposed a series of emission reduction measures in response to the greenhouse effect caused by carbon dioxide emissions. Among these, carbon capture, utilization, and storage (CCUS) has attracted widespread attention as a potential greenhouse gas reduction technology [1].

Carbon dioxide(CO_2_) sequestration technology achieves long-term isolation from the atmosphere by injecting CO_2_ into suitable deep geological formations [2]. In practice, challenges remain regarding ensuring the effective storage and safety of CO_2_ underground. Among the various stages of geological CO_2_ sequestration, the permeability of sandstone reservoirs directly affects the distribution, migration, and storage effectiveness of CO_2_. Different temperature and pressure conditions can lead to changes in the CO_2_ phase within the reservoir, thereby influencing the permeability characteristics of the sandstone. As gaseous CO_2_ transitions to a supercritical state, its flow properties undergo fundamental changes, imposing new requirements on the storage capacity and stability of the sandstone reservoirs.

The efficient injection of CO_2_ is a prerequisite for effective sequestration of CO_2_. Understanding the influence of CO_2_ phase changes on the permeability of reservoir rocks is an effective way to enhance sequestration efficiency. Scholars, both domestically and internationally, have conducted extensive research on the CO_2_ injection and flow processes in sandstone. Ni et al [3] studied the effect of CO_2_ injection on the permeability of coal reservoirs, finding that the impact of CO_2_ injection on permeability follows a trend of initial decrease followed by an increase. Niu [4] found that CO_2_ injection can reduce the mechanical properties of the coal matrix. Gao [5] established models for the relationship between porosity and permeability through CO_2_ injection experiments. Li et al. [6] studied the deformation of rocks under different coaxial confining pressures by establishing a two-dimensional rock constitutive model. Ju et al. [7] combined yield theory and determined the true tensile yield strength of sandstone through experimental methods. Wang et al. [8, 9] studied the energy distribution of coal and rock after CO_2_ fracturing and proposed a new damage calculation method after CO_2_ fracturing, which provided theoretical support for the stability of the system after CO_2_ fracturing. However, they did not consider the evolution of reservoir rock permeability after CO_2_ injection. Hao [10, 11] established an evolution equation for the permeability of coal under different pressure gradients by conducting coal adsorption experiments.Liu [12] discovered through continuous injection experiments of supercritical CO_2_ that continuous injection caused pore blockage, resulting in a 12.03% loss of porosity in the core samples. Liu [13] et al. constructed a microscopic gas-liquid permeation pathway using image analysis methods to evaluate the storage performance of CO_2_; Li et al. [14] studied the changes in gas concentration during the fracturing and displacement process of coal seams by injecting liquid CO_2_. Liu et al. [15, 16] and Sun et al. [17] studied the wettability of CO_2_ during reservoir flow. Bai et al. [18] studied the distribution law of formation stress under supercritical CO_2_ seepage based on finite element calculation method. Teng et al. [19] conducted experiments on CO_2_ permeation in coal under different stable pressures and discussed the sensitivity of confining pressure to permeability.

However, most existing studies focus on a single lithology or injection mode, lacking comparative analysis of different types of reservoirs (e.g., low permeability sandstone and high permeability sandstone). The influence of different geological conditions (e.g., stress field, porosity, etc.) on permeability may be significantly different, which is rarely discussed in the existing literature. Some studies [20] have ignored the influence of the stress state in the reservoir and CO_2_ injection interactions on permeability. In this study, CO_2_ injection experiments were conducted closer to the actual reservoir conditions (e.g., formation temperature, pressure, and stress state) to more accurately reflect the influence of CO_2_ injection on the permeability in an actual reservoir.

This study used sandstone from the Ordos Basin to conduct CO_2_ injection sandstone experiments and compare the changes in rock permeability under different water saturation levels. The focus was on studying the effects of injection pressure and saline concentration on reservoir rock permeability during CO_2_ injection to provide ideas for efficient CO_2_ storage.

## 2. Materials and methods

### 2.1. Sample preparation

**Fig 1:**
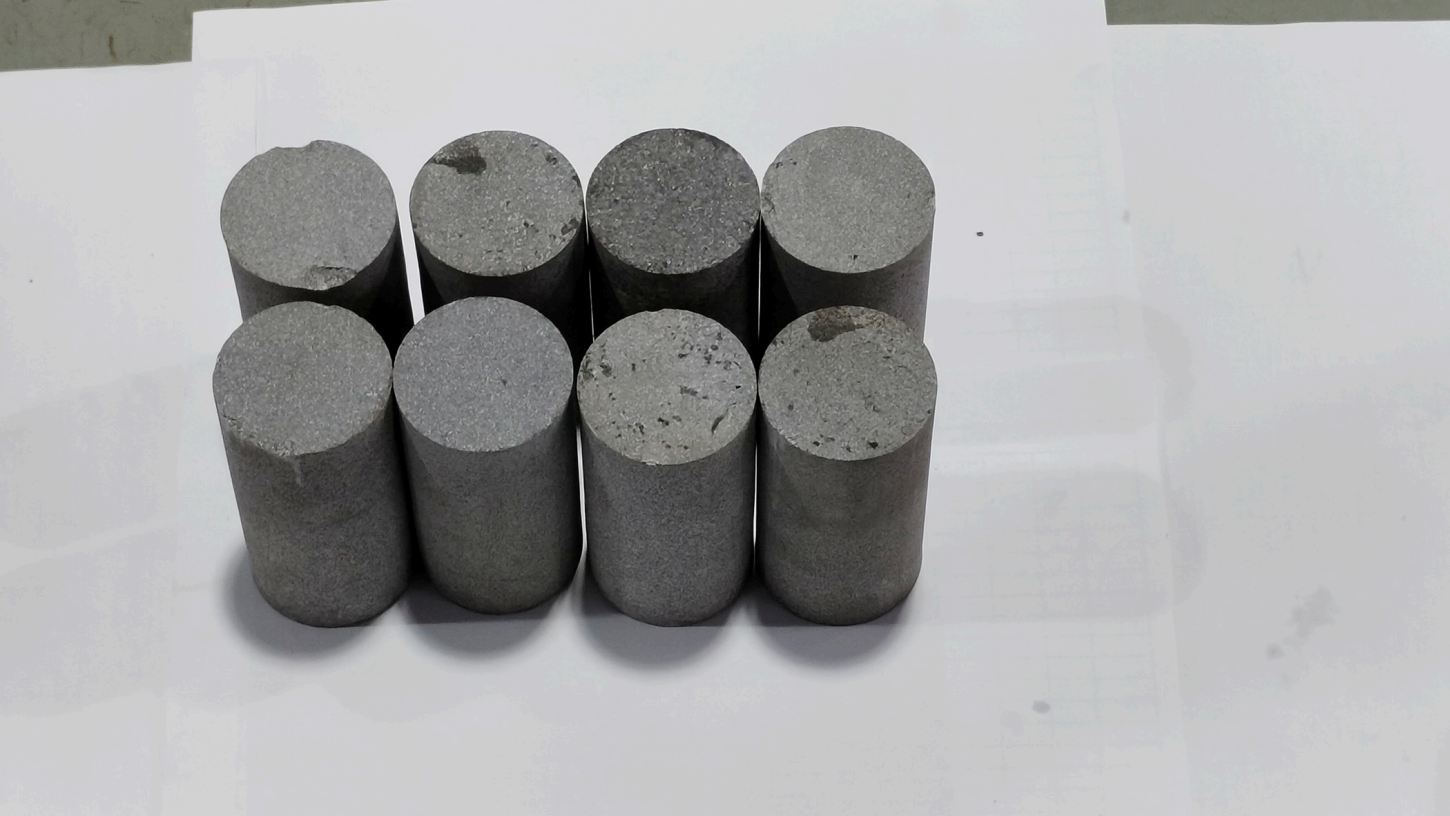
Sandstone sample diagram.

The experimental sandstone (Fig 1) was obtained from the National Energy Sensitive East No.1 Mine in the Hailar Development Zone, Hulunbuir City, Inner Mongolia Autonomous Region, at a burial depth of 850 m. According to the guiding principles of the International Society for Rock Mechanics (ISRM) [21], the core was processed into standard cylindrical specimens with diameters of 50 mm and heights of 100 mm to ensure a smooth end face.

**Fig 2:**
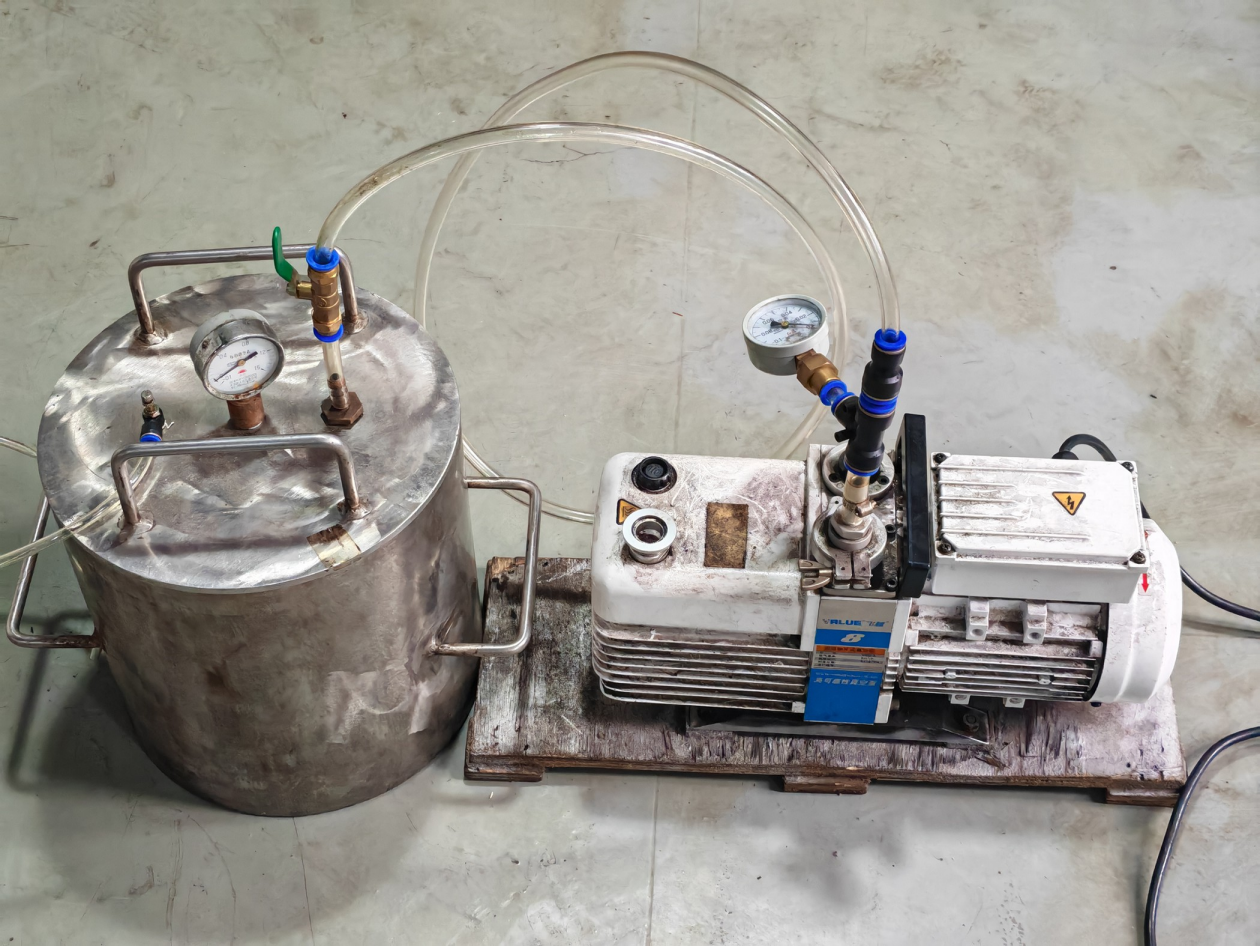
Vacuum saturation equipment.

**Fig 3:**
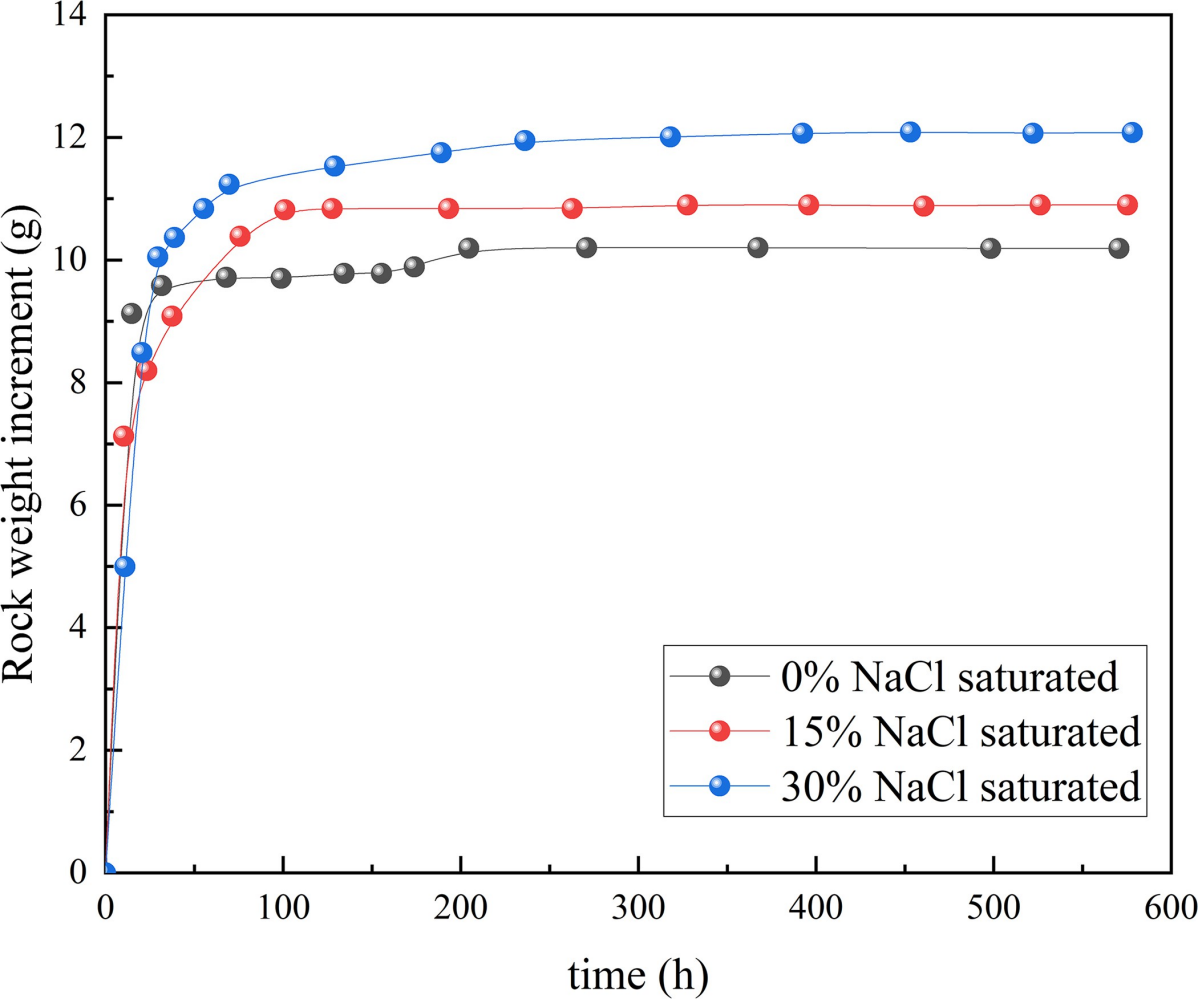
Time-dependent curves of sandstone quality immersed in different concentrations of salt water.

Three different concentrations of saltwater solutions, 0%, 15%, and 30% (saltwater mass ratio), were configured as vacuum-saturated (Fig 2) sandstone using a vacuum saturation device until the mass remained unchanged (Fig 3), and a set of dried sandstone was used as a control.

### 2.2. Test system

To accurately analyze the influence of phase changes on the permeability of sandstone during CO_2_ injection, an advanced seepage experimental device was designed and used to conduct steady-state seepage experiments based on the different injection effects of gaseous and supercritical CO_2_. The experimental setup mainly consisted of a high-pressure injection module, a constant temperature bath, a sandstone core gripper, a pressure and flow monitoring system, and a data acquisition unit. The high-pressure injection module can simulate the high-pressure environment deep in the formation, compress CO_2_ to a supercritical state, and ensure a stable CO_2_ pressure throughout the entire experimental process. A constant temperature bath was used to maintain a constant ambient temperature around sandstone cores. The axial and confining pressure loading systems in the self-developed equipment are provided by the twin-cylinder constant speed constant pressure pump TC-100D, with a maximum pressure can reach 70MPa, and a flow adjustment range of 0.01 ~ 30 ml/min. The performance was significantly better than that of the Quiznix Q5000 precision metering pump (Chandler Engineering) with a maximum pressure of 15MPa in Main et al. [22].

**Fig 4:**
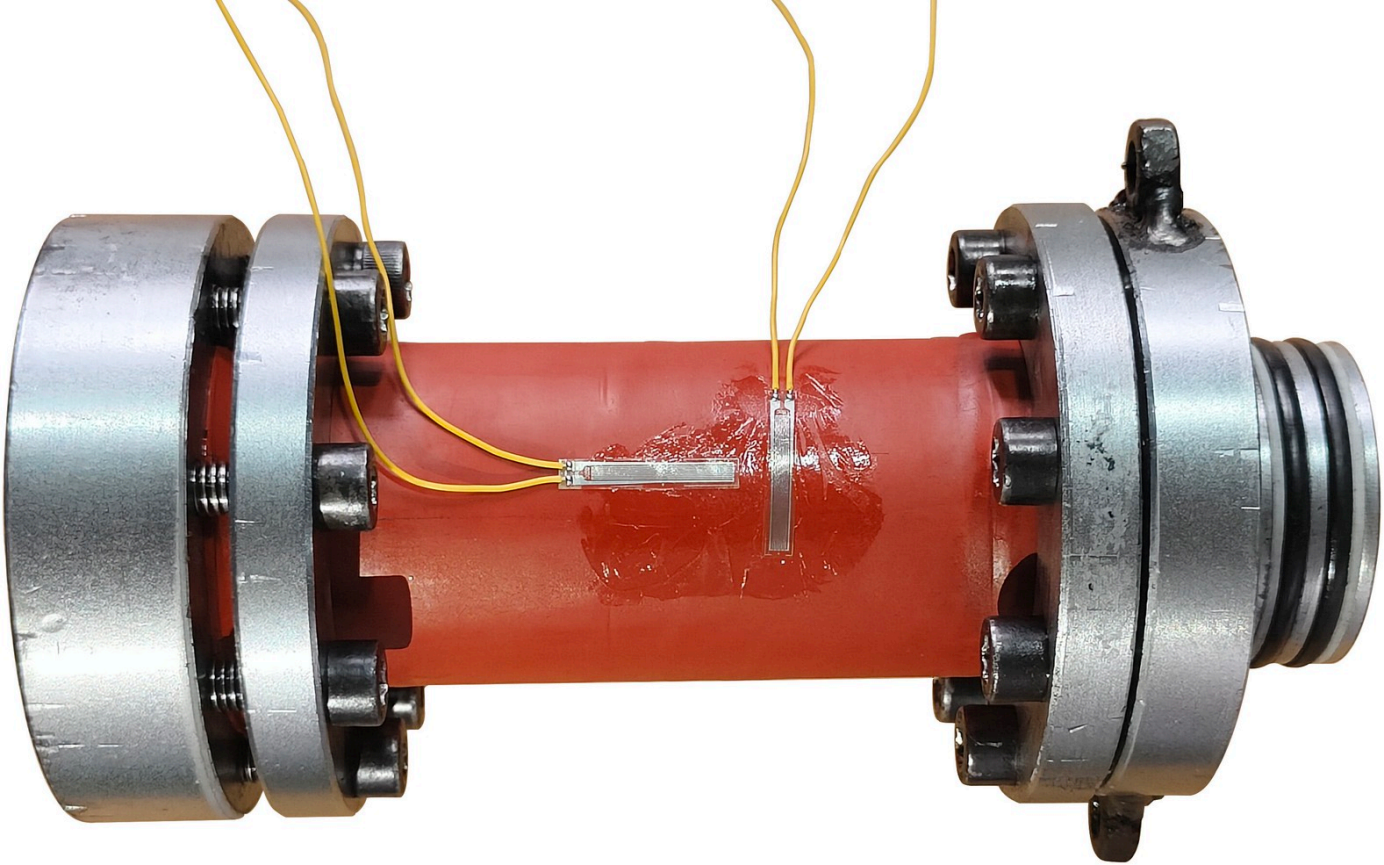
Layout of external strain gauges on rock cores.

The sandstone core holder is the core part of the experimental device, made of corrosion-resistant high-strength materials, capable of withstanding high-pressure injection of supercritical CO_2_, while ensuring the integrity of the core sample and achieving accurate permeability measurements. After the core was wrapped with Nitrile rubber heat shrink sleeve, a strain gauge was attached to the outside to measure the strain of the sandstone during the experiment. In order to isolate the strain gauge from the confining pressure loading medium oil, waterproof tape was tightly wrapped around the outside of the strain gauge. The arrangement of strain gauges is shown in Fig 4.

**Fig 5:**
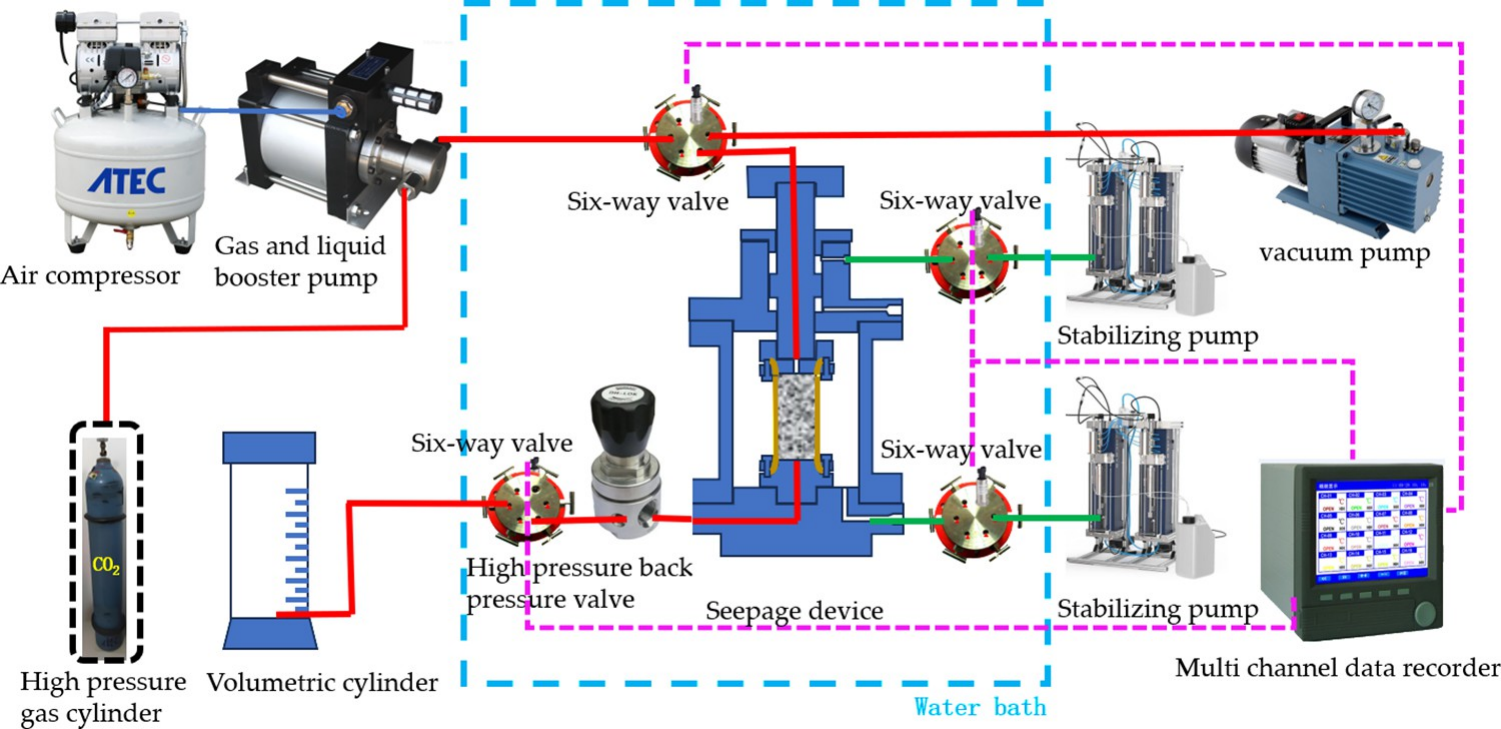
Schematic of seepage experimental device.

The entire gripper design required a high degree of sealing to eliminate the possibility of gas leakage and ensure the reliability of experimental data. The pressure and flow monitoring system monitored the pressure and CO_2_ flow status in real-time during the experimental process through high-precision sensors. The data acquisition unit was responsible for recording all relevant parameters, including pressure, temperature, and flow rate, for subsequent data analysis. The experimental setup is shown in Fig 5.

### 2.3. Experimental methods and data processing

#### 2.3.1. Experimental steps

In an experiment on the influence of CO_2_ geological storage on sandstone permeability, the permeability measurement process is a crucial link, which directly affects the accuracy and reliability of the experimental results.

The steady-state seepage experiment of the independently developed seepage device simulated the sandstone layer conditions at different depths of the geological environment by precisely controlling the injection pressure and temperature parameters, ensuring the realism and scientificity of the experiment. During the experiment, under simulated reservoir conditions, the sandstone sample was placed in a core clamp to ensure a sealed and high-pressure environment. Subsequently, CO_2_ was injected into the sandstone sample through a high-precision pressure pump system. To ensure the accuracy of the permeability data, the steady-state flow method was used to test the initial permeability of the experimental samples. The strata 850 m underground in Mindong No. 1 Mine belongs to the sandstone layer, and some areas contain salt water in the sandstone, which not only provides a huge space for CO_2_ geological storage, but also forms a good sealing condition. To study the seepage characteristics of the CO_2_ injection process, CO_2_ seepage experiments were conducted under different injection pressures and effective stresses. Ensure that the injection pressure can effectively promote CO_2_ injection without causing a reservoir fracture.

The experimental plans are listed in Table 1. Then injection pressure was gradually increased, and high-precision differential pressure sensors were used to record the pressure difference and flow rate changes, and monitor the real-time temperature of the experimental process through temperature sensors. Different temperature and pressure points were set according to the experimental requirements to simulate gas flow behavior at different depths and geological conditions. In addition, to reduce possible errors introduced by the experimental equipment, the seepage device was repeatedly calibrated and tested to ensure data reliability.

**Table 1:** Experimental parameters.

Sample status	Injection pressure/MPa	confining pressure/MPa	Effective stress/MPa
Dry rock 0% NaCl saturated rock 15% NaCl saturated rock 30% NaCl saturated rock	2	3→30	1→3→5→7→10→13→18→23→28
	4	5→32	
	6	7→34	
	8	9→36	
	10	11→38	

**Fig 6:**
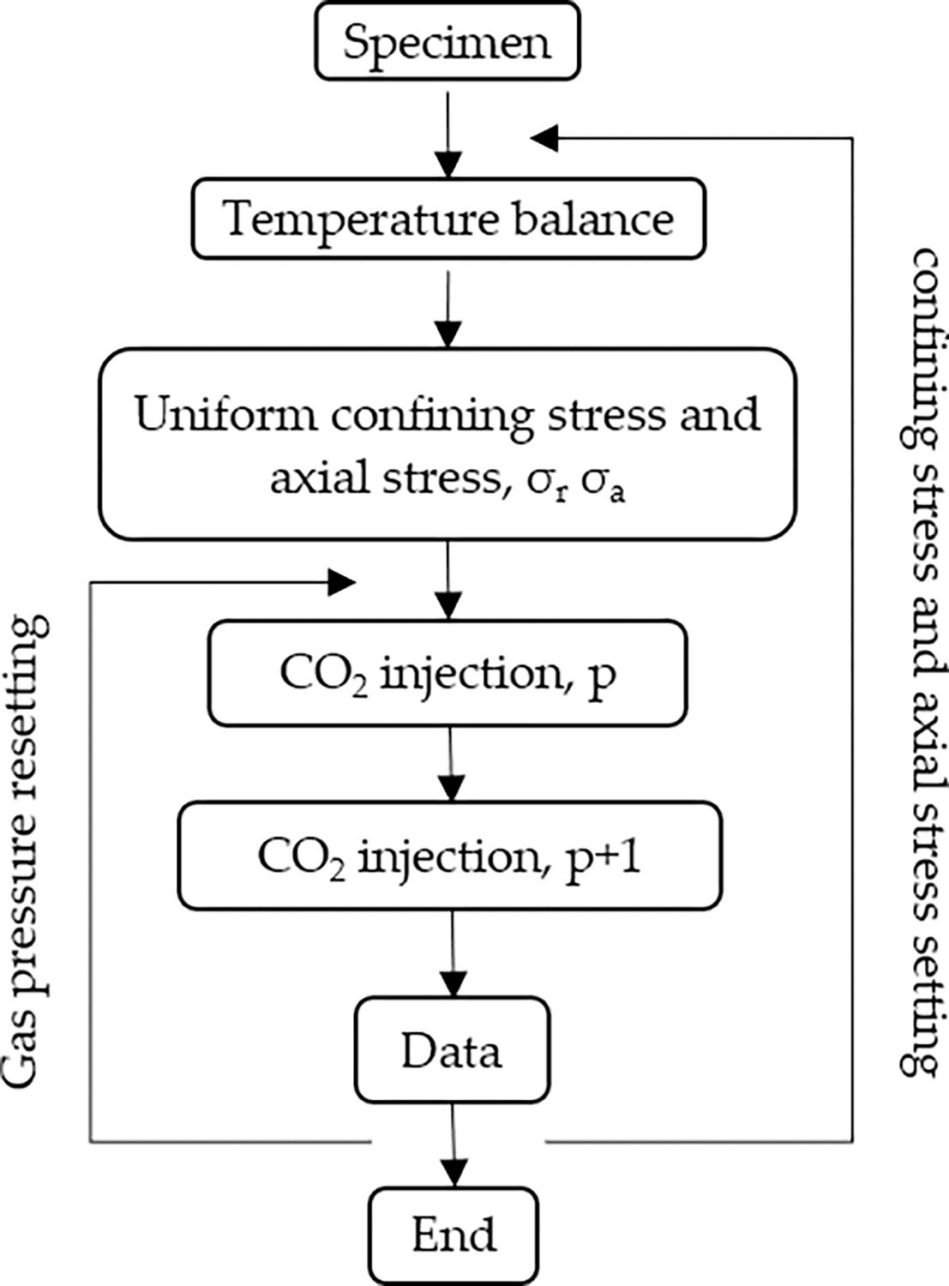
Experimental flowchart.

The experimental process is illustrated in Fig 6. The sample was placed in a three-axis cavity under a constant and uniform confining pressure. After the temperature of the entire device remained constant at 35°C, CO_2_ fluid at a predetermined pressure was injected into the permeation device for CO_2_ permeation experiments.

The sandstone core was placed in a triaxial chamber and a stress of 1 MPa was applied in the vertical direction. The lateral and vertical stresses were alternately increased in steps of 1 MPa until both reached a set pressure. Inert helium gas was injected at a constant pressure(0.5 MPa) to remove air from the rock core. During this process, a gas leak check was conducted in the system. A vacuum pump was then used to remove the residual helium gas from the entire system. Subsequently, CO_2_ was injected into the system at an initial pressure of 1 MPa. When the gas flow rate in the outlet section was stable, seepage was considered to have reached equilibrium. After reaching a new equilibrium, CO_2_ at a gas pressure of 2 MPa (1 MPa higher than the initial equilibrium pore pressure) was injected into the system for seepage until a new seepage equilibrium was reached. After completing the test under a CO_2_ pressure of 1 MPa, the experiment was conducted according to Table 1, by repeating the same procedure.

#### 2.3.2. Calculation of permeability

Accurate calculation of permeability is important to guarantee the accuracy of experimental data when conducting research on CO_2_ injection into sandstones. The key factors to be considered in the permeability calculation method include the fluid viscosity, injection pressure, porosity, and permeability characteristics of sandstone. The permeability of the sandstone can be obtained through a comprehensive analysis of these parameters. The calculation method was based on Darcy’s law to describe the infiltration flow, and the permeability of sand was determined based on the relationship between the flow rate and pressure drop.

The permeability was calculated using the following formula:

$$k=\frac{q_{ave}\mu_{ave}L}{A\Delta p}\times 10^{5}$$ (1)

Where k is the permeability, mD; *q*_*ave*_ is the average gas flow rate through the core calculated at the average pore pressure, cm^3^/s; *μ*_*ave*_ is the viscosity of the gas calculated at the average pore pressure of the experimental temperature, pa⋅s; L is the length of the rock core, cm; A is the cross-sectional area of the rock core, cm^2^; Δ*p* is the difference in gas pressure between the upstream and downstream of the rock core, *p*_1_−*p*_2_, MPa;

The relationship between the flow rates *q*_0_ and *q*_*ave*_ atmospheric pressure *p*_0_ is

$$\frac{(p_{1}+p_{2})q_{ave}}{2}=p_{0}q_{0}$$ (2)

The expression for gas permeability is

$$k=\frac{2\mu_{ave}p_{0}q_{0}L}{(p_{1}^{2}-p_{2}^{2})A}\times 10^{5}$$ (3)

In this study,the mass flow rate was used to calculate the permeability of gaseous and supercritical CO_2_ in the rock cores. The flow rate of CO_2_ is related to the flow rate of CO_2_ at the outlet atmospheric pressure as follows:

$$\bar{\rho }q_{ave}=\rho_{0}q_{0}$$ (4)

Where: $\bar{\rho }$ is the average density of gas in the rock core, kg⋅m^-3^, *q*_*ave*_ is the average flow rate of gas in the rock core, cm^3^/s; ${\bar{\rho }}_{0}$ and *q*_0_ are the gas density and flow rate at atmospheric pressure, respectively, kg⋅m^-3^, cm^3^/s

The formula for calculating the permeability of rock cores in different CO_2_ phases is as follows:

$$k=\frac{\mu_{ave}Lq_{0}}{A\Delta p}\cdot \frac{\rho_{0}}{\bar{\rho }}\times 10^{5}$$ (5)

For CO_2_ in different phases, the impact of phase changes on viscosity was also considered. During the experiment, temperature correction coefficients were established for both gaseous and supercritical CO_2_ to correct for the effect of temperature changes on the permeability calculations. Considering the intensified thermal motion of gas molecules at high temperatures and the special properties of supercritical CO_2_, determination of the correction coefficient plays a decisive role in reflecting the true permeability.

The AP1700 material property query platform provides a large number of basic physical property parameters that can be used in the industry, and its data are consistent with those published by the National Institute of Standards and Technology (NIST) in the United States. The experimental gas CO_2_ was selected to fit the relationship between gas viscosity, temperature, and pressure.

**Fig 7:**
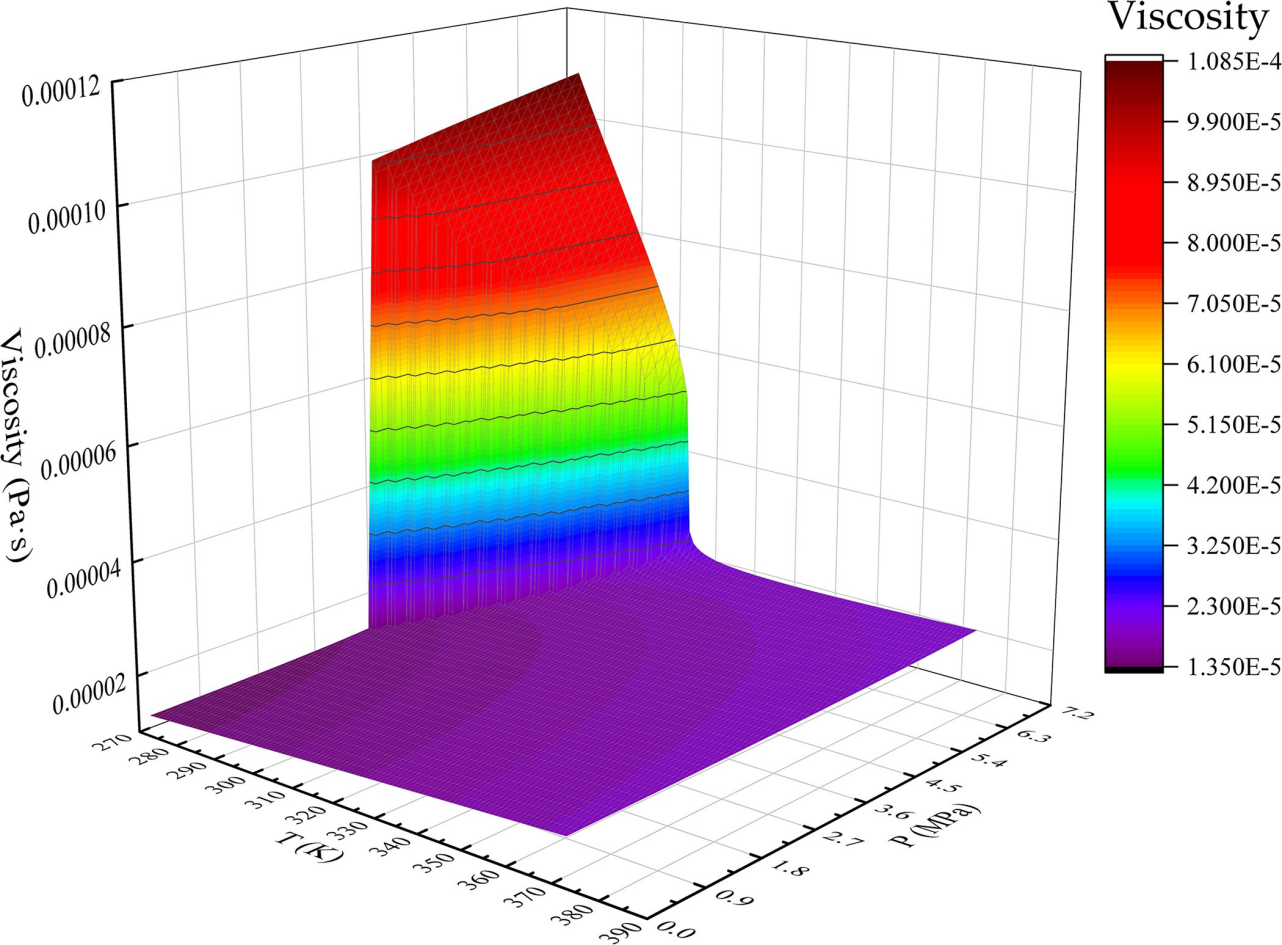
0~7.38 MPa, 273.15 K~373.15 K CO_2_ viscosity temperature pressure relationship surface.

**Fig 8:**
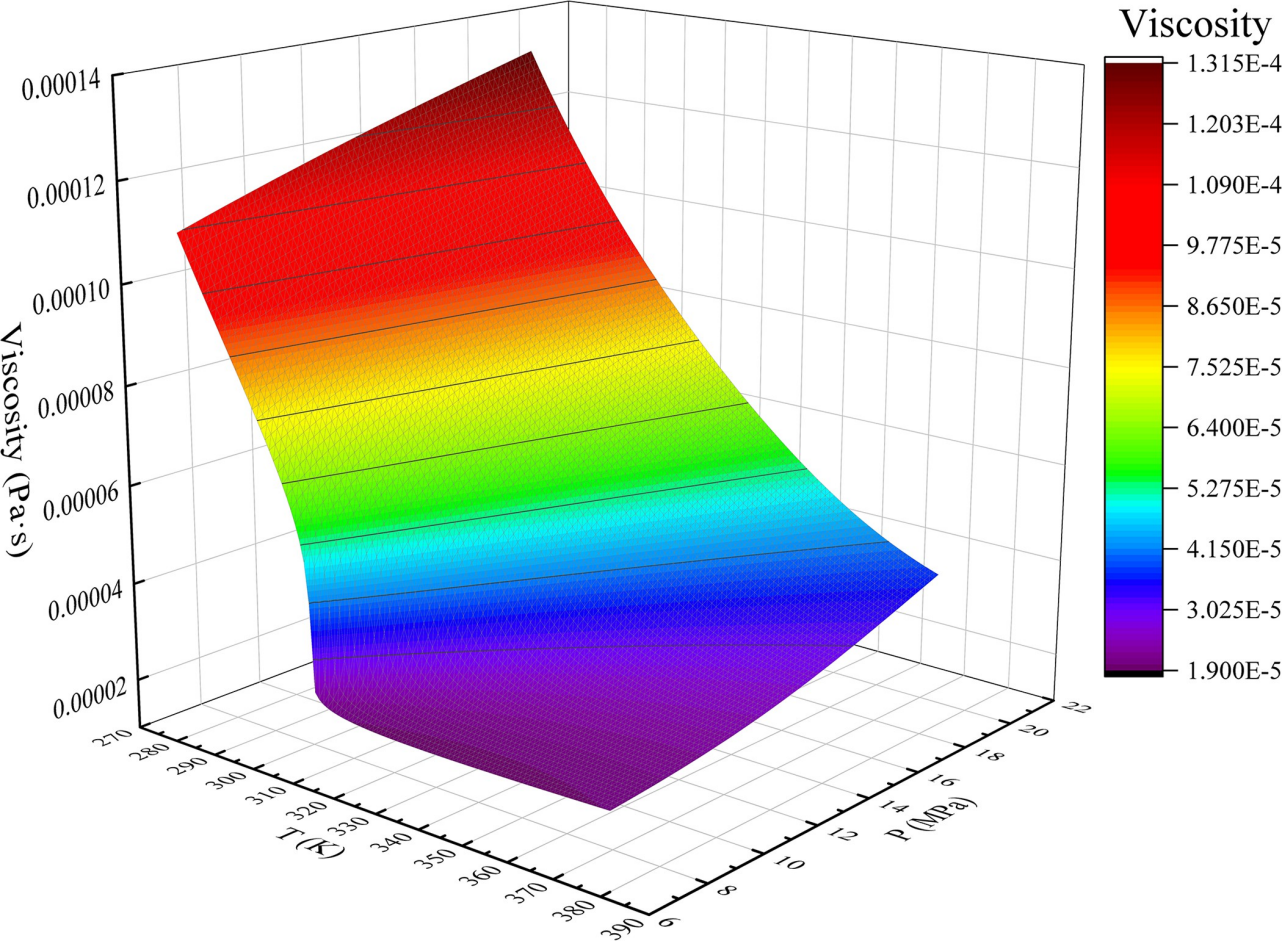
7.38 MPa~20 MPa, 273.15 K~373.15 K CO_2_ viscosity temperature pressure relationship surface.

A graph was created after retrieving the CO_2_ physical property parameters using the AP1700 material property query platform. Fig 7 shows the viscosity temperature pressure relationship surface graph from 0 to 7.38 MPa, 293.15 K to 373.15 K, and Fig 8 shows the viscosity temperature pressure relationship surface graph from 7.38 MPa to 20 MPa, 273.15 K to 373.15 K.

A polynomial expression was obtained by fitting the data using the least-squares method. As CO_2_ is at a critical pressure of 7.38 MPa, the fitting region was divided into 0~7.38 MPa, 293.15 K~373.15 K, and 7.38 MPa~20 MPa, 273.15 K~373.15 K. The fitting is a fifth degree polynomial, and the viscosity calculation accuracy meets the requirements.

The temperature-pressure-viscosity relationship of CO_2_ below the critical pressure is

$$\begin{array}{l} f(\mu_{g})=f(T,P)=\\ \;a_{1}+a_{2}T+a_{3}P+\\ \;a_{4}T^{2}+a_{5}TP+a_{6}P^{2}+\\ \;a_{7}T^{3}+a_{8}T^{2}P+a_{9}TP^{2}+a_{10}P^{3}+\\ \;a_{11}T^{4}+a_{12}T^{3}P+a_{13}T^{2}P^{2}+a_{14}TP^{3}+a_{15}P^{4}+\\ \;a_{16}T^{5}P+a_{17}T^{4}P+a_{18}T^{3}P^{2}+a_{19}T^{2}P^{3}+a_{20}T^{4}P+a_{21}P^{5} \end{array}$$ (6)

*μ*_*g*_ is the viscosity of gaseous, pa⋅s; *T* is the gas temperature, K; *P* is the gas pressure, MPa.

The temperature-pressure-viscosity relationship of CO_2_ above the critical pressure is

$$\begin{array}{l} f(\mu_{g-Sc})=f(T,P)=\\ \;b_{1}+b_{2}T+b_{3}P+\\ \;b_{4}T^{2}+b_{5}TP+b_{6}P^{2}+\\ \;b_{7}T^{3}+b_{8}T^{2}P+b_{9}TP^{2}+b_{10}P^{3}+\\ \;b_{11}T^{4}+b_{12}T^{3}P+b_{13}T^{2}P^{2}+b_{14}TP^{3}+b_{15}P^{4}+\\ \;b_{16}T^{5}P+b_{17}T^{4}P+b_{18}T^{3}P^{2}+b_{19}T^{2}P^{3}+b_{20}T^{4}P+b_{21}P^{5} \end{array}$$ (7)

The polynomial coefficients in Formulas (6) and (7) are shown in Tables 2 and 3.

**Table 2:** CO_2_ viscosity temperature pressure relationship coefficient a.

coefficient	value	coefficient	value	coefficient	value
a_1_	-1.35E-01	a_8_	-7.21E-08	a_15_	-1.90E-06
a_2_	2.17E-03	a_9_	2.69E-06	a_16_	4.72E-14
a_3_	1.20E-03	a_10_	1.03E-10	a_17_	-3.63E-13
a_4_	-1.40E-05	a_11_	-7.32E-11	a_18_	1.34E-11
a_5_	1.12E-08	a_12_	3.05E-10	a_19_	-2.10E-10
a_6_	-2.49E-04	a_13_	-1.04E-08	a_20_	4.45E-09
a_7_	4.53E-08	a_14_	7.86E-08	a_21_	2.21E-08

**Table 3:** CO_2_ viscosity temperature pressure relationship coefficient b.

coefficient	value	coefficient	value	coefficient	value
b_1_	7.96E-12	b_8_	-4.84E-10	b_15_	1.21E-08
b_2_	7.18E-10	b_9_	-1.34E-09	b_16_	6.29E-16
b_3_	-1.73E-11	b_10_	8.53E-12	b_17_	-1.55E-15
b_4_	9.36E-09	b_11_	-3.55E-13	b_18_	-3.74E-13
b_5_	3.79E-11	b_12_	3.17E-12	b_19_	9.10E-12
b_6_	8.04E-10	b_13_	7.33E-11	b_20_	-8.46E-11
b_7_	8.53E-12	b_14_	-1.67E-09	b_21_	2.19E-10

By substituting (6) and (7) into (5) respectively, we can obtain the CO_2_ sandstone seepage model considering the temperature gas pressure effect

$$\begin{array}{l} k_{g}=\frac{f(\mu_{g})Lq_{0}}{A\Delta p}\cdot \frac{\rho_{0}}{\bar{\rho }}\times 10^{5}\\ p\in (0MPa,7.38\;MPa),K\in (273.15\;K,373.15\;K)\\ k_{g-Sc}=\frac{f(\mu_{g-Sc})Lq_{0}}{A\Delta p}\cdot \frac{\rho_{0}}{\bar{\rho }}\times 10^{5}\\ p\in (7.38\;MPa,20\;MPa),\;K\in (273.15\;K∼373.15\;K) \end{array}$$ (8)

The flow characteristics of CO_2_ in sandstone media have a direct impact on the safety and efficiency of CO_2_ geological storage. A new relationship between the temperature and supercritical CO_2_ viscosity was introduced, and the model parameters were optimized through multiple iterations to achieve an accurate mathematical expression of the viscosity temperature pressure.

## 3. Results

### 3.1. The influence of saltwater saturation on the permeability of sandstone injected with CO_2_

**Fig 9:**
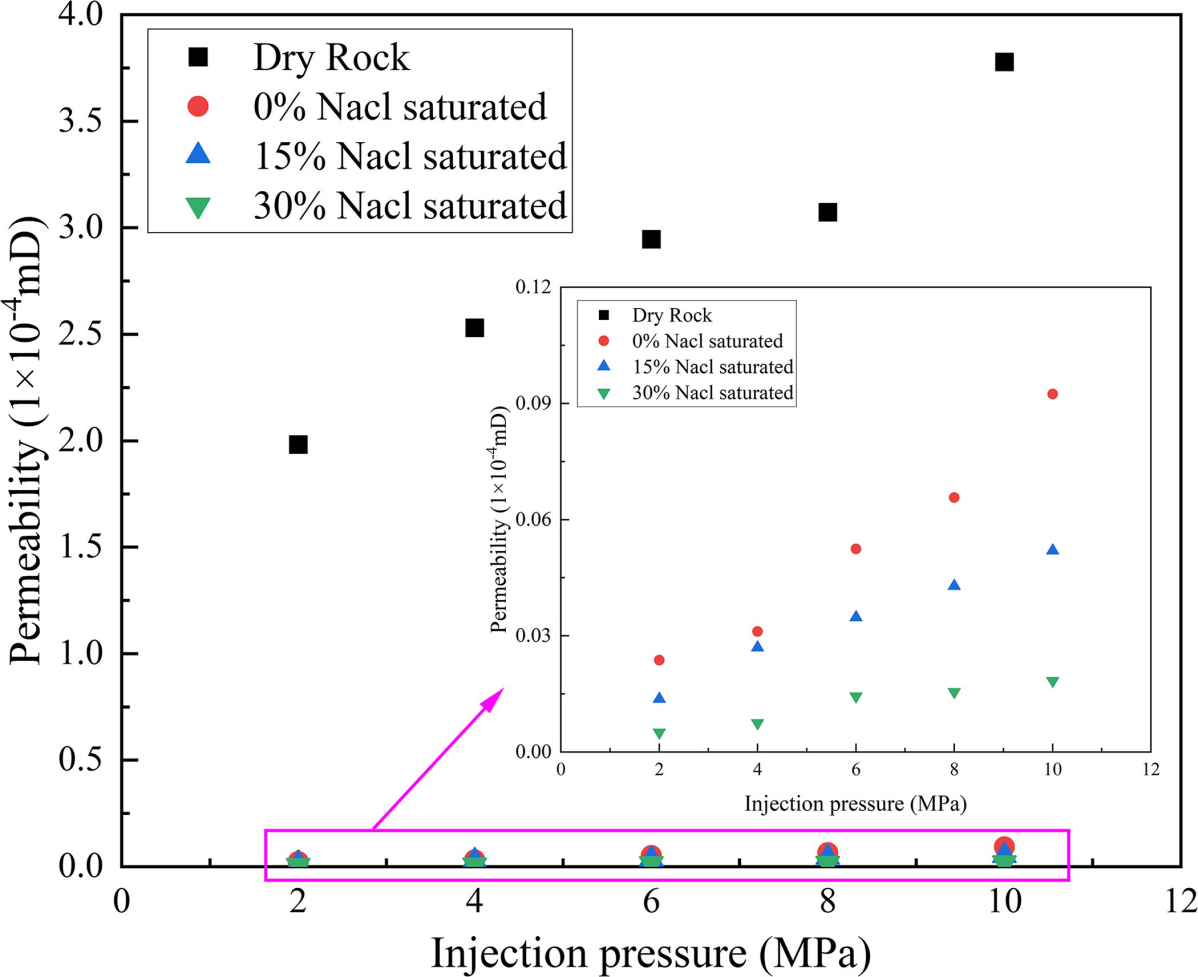
The relationship between different CO_2_ injection pressures and sandstone permeability (confining pressure = 20 MPa).

**Fig 10:**
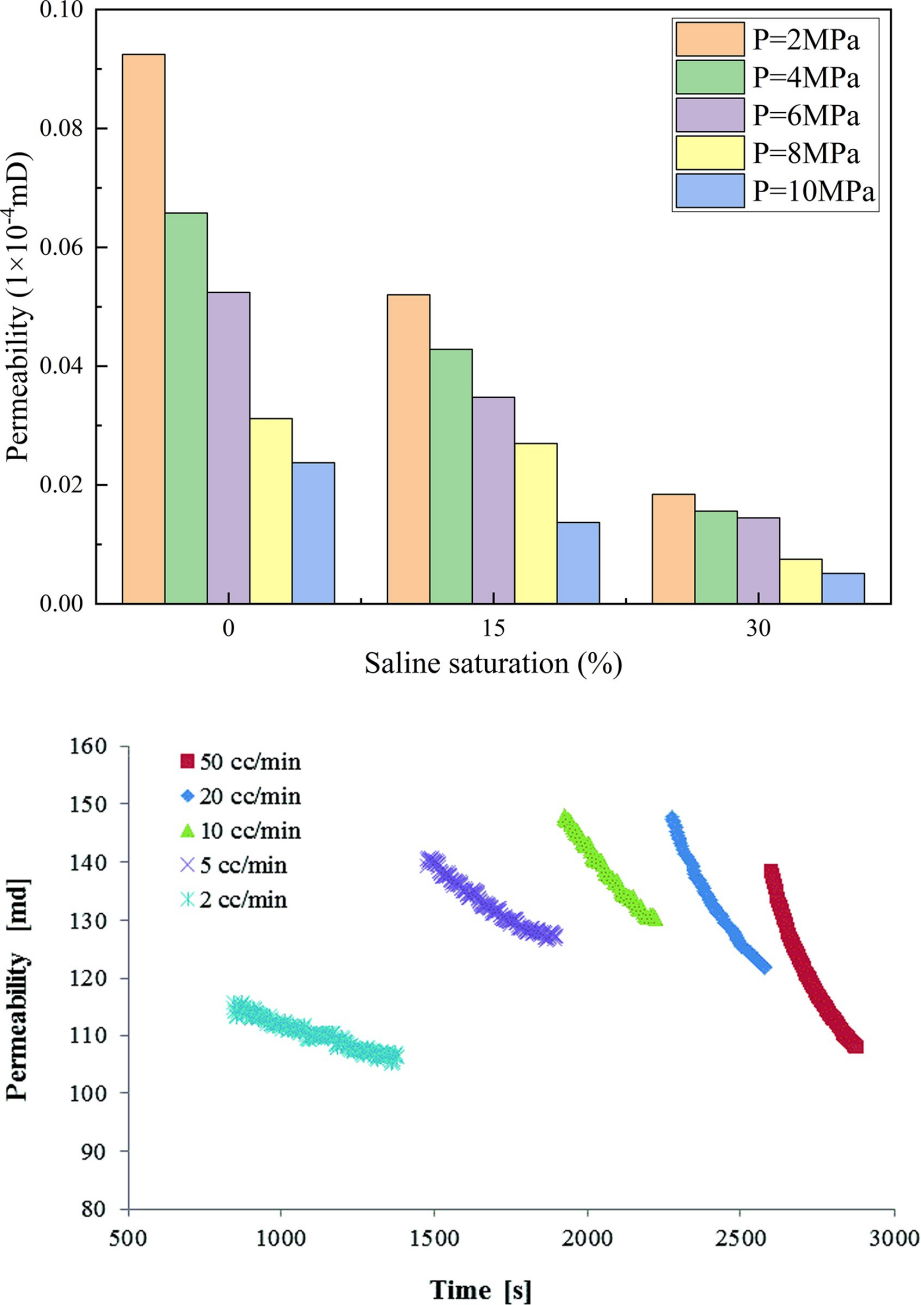
The relationship between saltwater saturation and permeability. (a) Variation in sandstone permeability. (b) Variation in Berea sandstone permeability reported by Iglauer et al. [23].

Fig 9 shows the relationship between the saltwater concentration and permeability evolution of the sandstone samples. After saltwater immersion, the permeability of the sandstone samples was approximately two orders of magnitude lower than that of the dry-rock samples. Fig 10(A) shows the relationship between the saltwater concentration and permeability. Fig 10(B) shows the change in permeability of the Berea samples studied by Glauer et al. [23]. The decrease of rock permeability after brine immersion was the same as the decrease of sandstone permeability from 1.60mD to 1.02mD after CO_2_ action conducted by Zhang et al. [24] in the laboratory.

**Fig 11:**
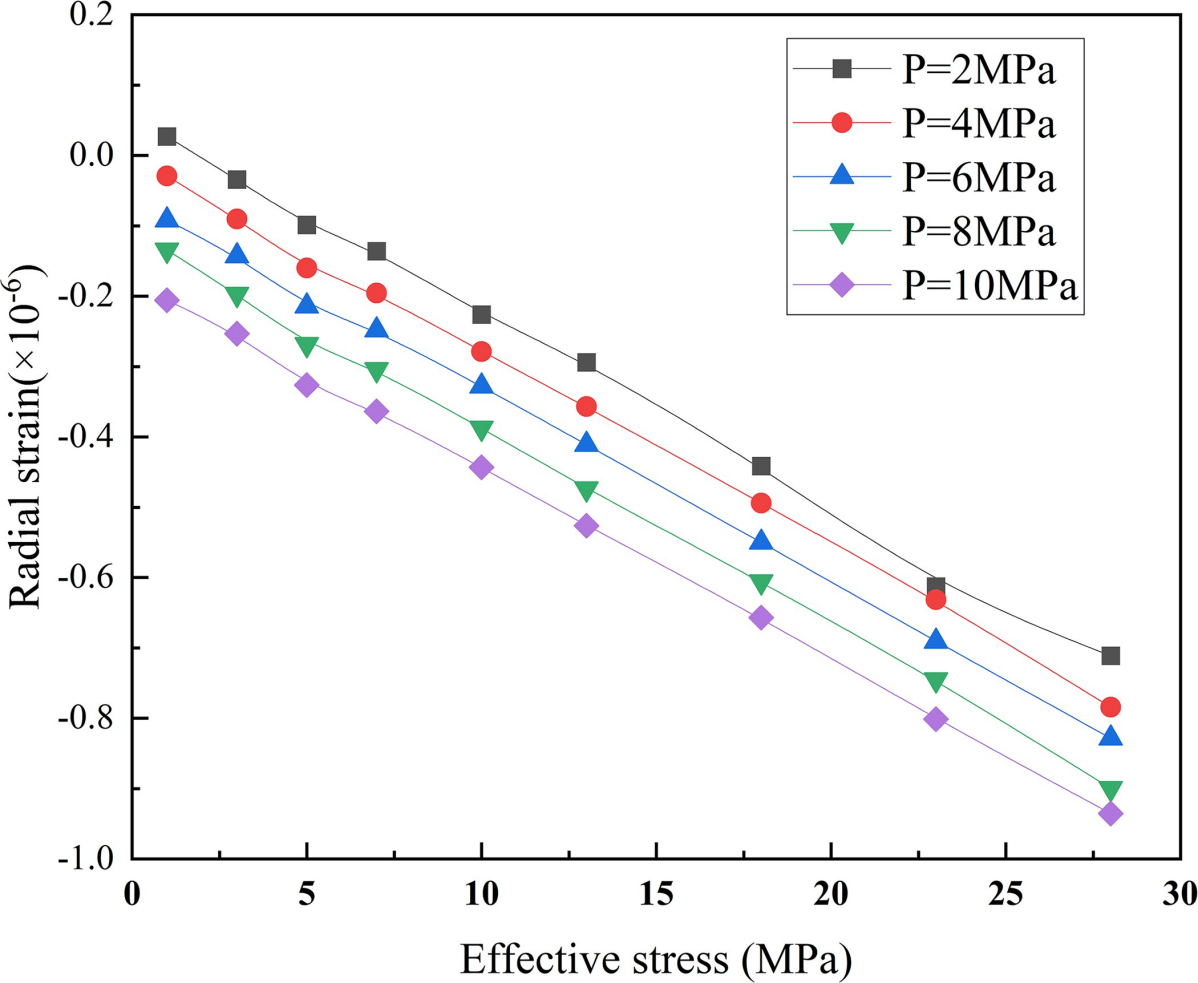
Sandstone radial strain under different effective stresses.

This phenomenon is related to the NaCl crystal content in the pore structure of the sandstone samples under different saltwater concentrations. Influenced by NaCl crystals, as the saltwater concentration increased, the degree of crystallization in the internal pores of the rock sample increased, which to some extent blocked the permeability channels, reduced the permeability channels, and led to a decrease in permeability. This is consistent with the research results of Akindipe et al. [25] that with the injection of CO_2_, salt in rock pores began to crystallize, and these crystalline substances blocked rock pores, resulting in a significant decrease in permeability after CO_2_ injection leads to an increase in water saturation, an increase in breakthrough pressure, and a decrease in the mechanical strength and elastic properties of the rock. These changes have an important impact on both the injectivity and storage capacity of CO_2_ and must be fully considered in CO_2_ geological storage projects [26, 27].When the CO_2_ injection pressure was 4MPa, the permeability of the dried sample was 81.2 times, 93.8 times, and 336.9 times that of the sandstone saturated with different concentrations of saline water. As the saline water concentration increased, the sandstone was subjected to stress during the initial stage of confining pressure loading, causing the pores to close and the saline filling between the cracks to be compressed, resulting in the blockage of the seepage channel and significant deformation of the cracks. The opening of the cracks decreased significantly, leading to a reduction in the seepage channels and permeability. Moreover, as the saline-water concentration increased, the blocking effect became more pronounced. Fig 11 shows the variation trend of the radial strain of the sandstone under different effective stresses.

### 3.2. The effect of effective stress on the permeability of saturated sandstone injected with CO_2_

**Fig 12:**
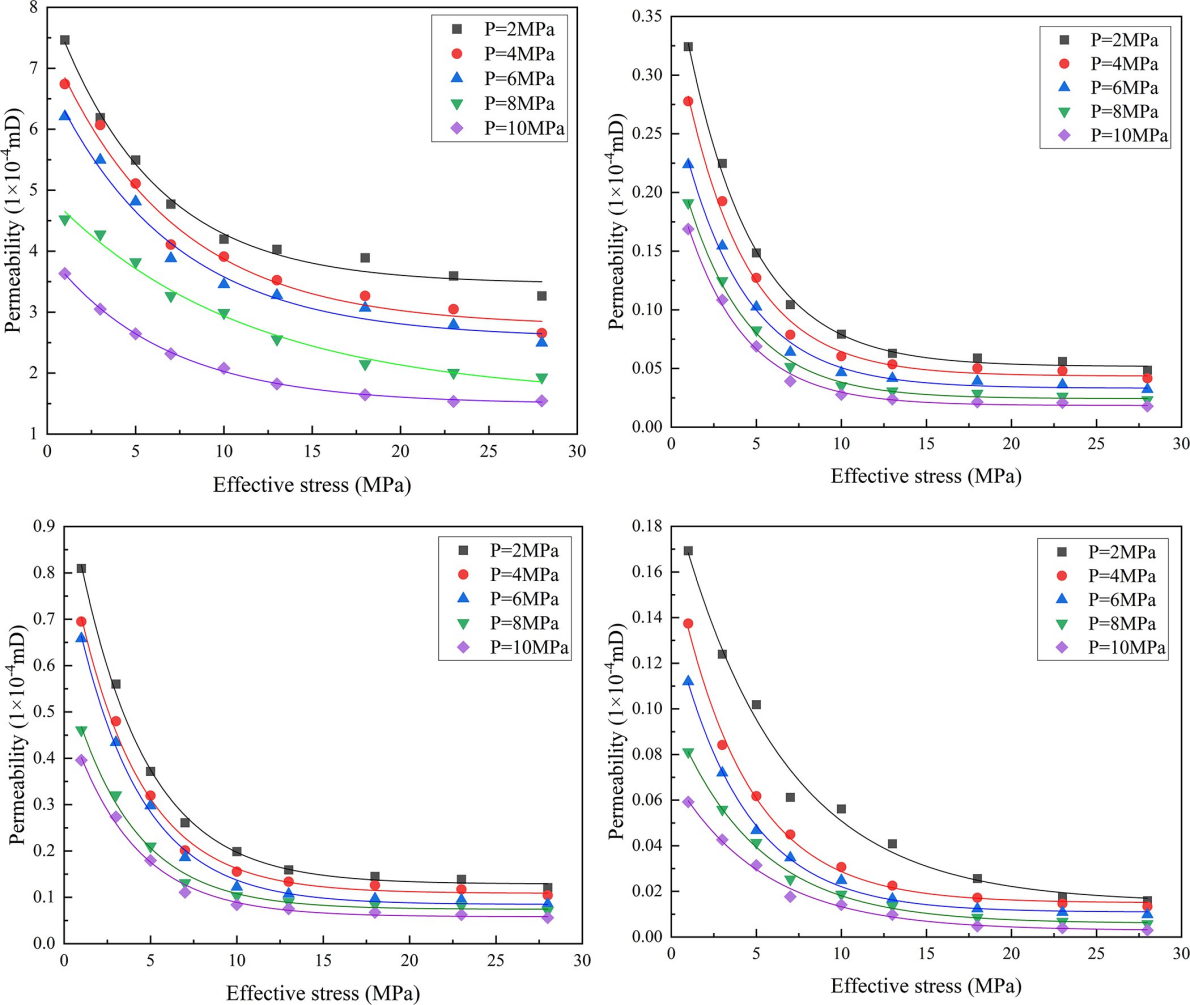
Relationship curve between effective stress and permeability of sandstone. (a) Dry rock; (b) 0%NaCl saturated; (c) 15%NaCl saturated; (d) 30%NaCl saturated.

Fig 12 shows the relationship between the effective stress and permeability of sandstone during CO_2_ injection when saturated with different concentrations of NaCl. Under the same effective stress path conditions, the permeability of sandstone gradually decreased with an increase in osmotic pressure. This trend was generally observed under different effective stress conditions, which is consistent with the results of Wang et al. [28]. When the osmotic pressure was constant, the permeability of the sandstone gradually decreased with an increase in the effective stress and eventually plateaued. Ye et al. [29] and Nasvi et al. [30] showed in CO_2_ injection experiments that increasing the effective stress in sandstone would lead to pore compression and subsequent permeability reduction. This phenomenon is critical for understanding the behavior of CO_2_ in geologic storage and its impact on reservoir storage and injection capacity because the sandstone samples contained a large number of pores and fractures in the rock mass during the initial stress loading stage. As the effective stress increases from 1MPa to 7MPa, the compaction degree of the sample increased, and the pores and fractures were compressed. The diameters of the effective pores and seepage channels become increasingly smaller, resulting in a sharp decrease in permeability. As the effective stress continued to increase, the pore and fracture volumes of the sandstone were further compressed. When the effective stress was loaded to 13 MPa, the decrease in permeability slowed, mainly because the increase in confining pressure gradually reduced the compression effect of the pores and permeable channels. The diameter of the effective pores and permeable channels tends to be stable; therefore, the change in sandstone permeability gradually becomes flat.

Tables 4–7 show the decrease in permeability when the effective stress of sandstone increases under different conditions.

**Table 4:** The decrease in sandstone permeability under different injection pressures as effective stress increases (Dry Rock).

Effective stress path/	injection pressure /MPa				
MPa	2	4	6	8	10
1→3	17.11%	9.99%	11.48%	5.47%	16.08%
3→5	11.20%	15.82%	12.43%	10.65%	13.31%
5→7	13.15%	19.57%	19.34%	14.51%	12.36%
7→10	12.03%	4.82%	10.97%	8.48%	10.26%
10→13	4.01%	9.87%	5.16%	14.57%	12.38%
13→18	3.44%	7.30%	6.34%	15.89%	9.78%
18→23	7.63%	6.67%	9.03%	6.63%	6.63%
23→28	9.09%	12.99%	10.64%	3.76%	-0.65%
Average increase amplitude	9.71%	10.88%	10.67%	9.99%	10.02%

**Table 5:** The decrease in sandstone permeability under different injection pressures as effective stress increases (0%NaCl saturated).

Effective stress path/	injection pressure /Mpa				
Mpa	2	4	6	8	10
1→3	30.82%	30.95%	30.95%	30.39%	30.87%
3→5	33.64%	33.42%	34.45%	34.52%	34.42%
5→7	29.79%	37.05%	37.61%	37.58%	38.30%
7→10	23.90%	22.78%	34.25%	21.36%	24.14%
10→13	19.87%	13.93%	12.50%	12.35%	10.61%
13→18	8.80%	5.71%	9.52%	7.04%	10.17%
18→23	4.39%	7.07%	2.63%	3.03%	7.55%
23→28	12.84%	10.87%	9.46%	12.50%	10.20%
Average increase amplitude	20.51%	20.22%	21.42%	19.85%	20.78%

**Table 6:** The decrease in sandstone permeability under different injection pressures as effective stress increases (15%NaCl saturated).

Effective stress path/	injection pressure /Mpa				
Mpa	2	4	6	8	10
1→3	30.69%	30.66%	30.97%	34.72%	35.78%
3→5	33.92%	33.93%	33.65%	33.73%	36.53%
5→7	29.67%	38.13%	37.68%	37.72%	43.17%
7→10	24.17%	23.27%	27.13%	31.73%	29.11%
10→13	20.63%	11.48%	10.64%	12.68%	14.29%
13→18	6.30%	5.92%	5.95%	6.45%	10.42%
18→23	5.04%	4.54%	7.59%	8.62%	2.33%
23→28	13.27%	13.40%	10.96%	11.32%	14.29%
Average increase amplitude	20.46%	20.17%	20.57%	22.12%	23.24%

**Table 7:** The decrease in sandstone permeability under different injection pressures as effective stress increases (30%NaCl saturated).

Effective stress path/	injection pressure /Mpa				
Mpa	2	4	6	8	10
1→3	26.78%	38.74%	35.73%	31.09%	27.96%
3→5	17.83%	26.63%	35.05%	25.97%	26.27%
5→7	39.86%	27.22%	25.62%	38.78%	43.74%
7→10	8.31%	31.73%	28.57%	26.12%	20.13%
10→13	27.21%	26.55%	32.81%	27.20%	30.61%
13→18	37.37%	23.69%	25.91%	37.33%	51.88%
18→23	31.80%	14.76%	12.30%	20.78%	16.02%
23→28	8.70%	8.63%	9.32%	14.94%	25.50%
Average increase amplitude	24.73%	24.74%	25.66%	27.78%	30.26%

For example, when the osmotic pressure is 2MPa, the average decrease in permeability of dry sandstone is 9.71% as the effective stress increases from 1MPa to 28MPa; The average decrease in permeability of 0% saline saturated sandstone is 20.51%; The average decrease in permeability of sandstone saturated with 15% saltwater is 20.46%; The average decrease in permeability of sandstone saturated with 30% saline solution is 24.73%. As the concentration of saltwater increased, the permeability decreased further under conditions of increased stress. This is because the blocking effect of saltwater weakens the flow channel, resulting in different effects of saltwater concentration on permeability. Under the coupling effect of stress and saltwater, the permeability decreased further. As the effective stress continues to increase, the compaction effect of pores and cracks gradually weakens, and the decrease rate of permeability relative to the initial stage slows down owing to the influence of saltwater concentration. Therefore, under the same osmotic pressure, the effective stress directly affected the permeability of the rock sample. As the effective stress increased, the permeability decreased, and the rate of decrease slowed down. At the same time, the difference in permeability between different concentrations of salt water saturated states and dry rock samples gradually decreases.

### 3.3. The influence mechanism of different concentrations of saline saturation on the permeability characteristics of sandstone

The increase in effective stress directly causes compression of rock fractures, resulting in a decrease in permeability. CO_2_ sequestration often occurs in deep geological formations, where injected CO_2_ is in a supercritical state. The relationship between permeability and effective stress is as follows [31]:

$$k=k_{0}e^{-3C_{f}(\sigma -\sigma_{0})}$$ (9)

Where *k* is sandstone permeability, mD; *k*_0_ is the initial permeability of sandstone, mD; *σ* is effective stress, MPa; *σ*_0_ is the initial effective stress, MPa; *C*_*f*_ is the compression coefficient of sandstone fractures, MPa^-1^。

Eq (9) can be transformed into [32]:

$$C_{f}=\frac{\ln(\frac{k}{k_{0}})}{-3(\sigma -\sigma_{0})}$$ (10)

Using Eq (10), the fitting curves of the horizontal (*σ*−*σ*_0_) and vertical axes $-1/3\;\ln(\frac{k}{k_{0}})$ for a CO_2_ injection pressure of 8 MPa were obtained, where the slope of the curve is the compression coefficient C_f_ of the sandstone fractures.

**Fig 13:**
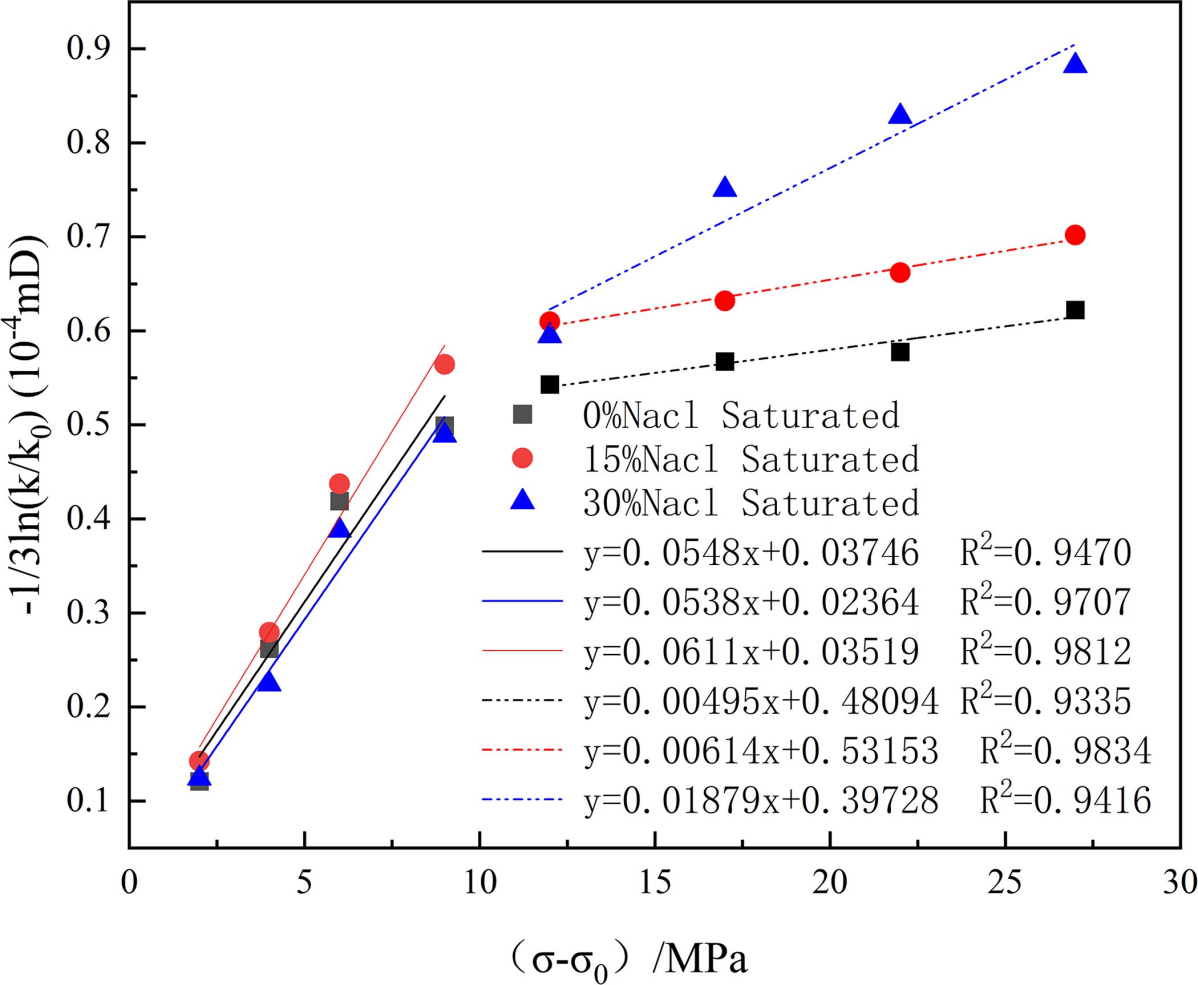
Curves of cleat compressibility computation.

Fig 13 shows that sandstone with different water saturation levels was more prone to fracture compression during the initial compaction stage of 0–10 MPa (0.0538 MPa^-1^~0.0611 MPa^-1^). Although the initial permeability of each sample was different, their compression coefficients differed very little: when the effective stress increased from 10 to 28 MPa, the fracture compression coefficients under the action of 0%, 15%, and 30% saltwater concentrations were 0.00495 MPa^-1^, 0.00614 MPa^-1^, and 0.01879 MPa^-1^. Under the same effective stress, the fracture compression coefficient increased with an increase in the saturated salt water concentration.

The function of effective stress is to cause compression effect in rock pores, thereby reducing the flow channels and lowering the permeability of sandstone. At the beginning of loading, the particles inside the sandstone were linearly compacted and underwent elastic deformation. When the loading stress increased to the rough contact strength of the particles, the rough contact was crushed and consolidated and the crushed particles began to fill the fracture space.

Permeability is also affected by the saltwater concentration. When the sandstone was saturated with saltwater, saltwater particles occupied the pore space of the sandstone. The higher the saltwater concentration, the more salt crystals there are inside the sandstone, and the more obvious the blocking effect, resulting in a decrease in permeability.

## 4. Conclusions

1. There is a good power function relationship between the permeability of reservoir sandstone and effective stress. During CO_2_ injection into the reservoir sandstone, the permeability of dry sandstone was two orders of magnitude higher than that of saturated sandstone.
2. Injecting 8MPa CO_2_ pressure into reservoir sandstone, the compression coefficient of sandstone fractures increases with the increase of effective stress from 10MPa to 28MPa.
3. The permeability of reservoir sandstone is affected by both effective stress and saturated saline concentration. The effective stress reduces the volume of the sandstone pores and fractures, resulting in a decrease in permeability. High-concentration saline produces a blocking effect by forming crystals inside sandstone fractures, whereas increasing the compression coefficient of sandstone fractures leads to a decrease in permeability.
